# Imaging the Alternatively Spliced D Domain of Tenascin C in a Preclinical Model of Inflammatory Bowel Disease

**DOI:** 10.1007/s11307-022-01758-6

**Published:** 2022-07-29

**Authors:** Liang Zhang, Yuzhen Wang, Kristoff T. Homan, Stephanie M. Gaudette, Andrew J. McCluskey, Ying Chan, Joanne Murphy, Mary Abdalla, Christine M. Nelson, Victor Z. Sun, Jamie E. Erickson, Heather L. Knight, Anca Clabbers, Annette J. Schwartz Sterman, Soumya Mitra

**Affiliations:** 1 AbbVie Bioresearch Center, 100 Research Dr, Worcester, MA 01605 USA; 2 Bristol Myers Squibb, 100 Binney St, Cambridge, MA 02142 USA; 3 Worcester Technical High School, 1 Officer Manny Familia Wy, Worcester, MA 01605 USA

**Keywords:** Inflammatory bowel disease, Near infrared molecular imaging, ECM imaging, Targeted delivery

## Abstract

**Purpose:**

To image colon-expressed alternatively spliced D domain of tenascin C in preclinical colitis models using near infrared (NIR)-labeled targeted molecular imaging agents.

Procedures.

A human IgG1 with nanomolar binding affinity specific to the alternatively spliced D domain of tenascin C was generated. Immunohistochemistry identified disease-specific expression of this extracellular matrix protein in the colon of mice given dextran sulfate sodium in the drinking water. The antibody reagent was labeled with the NIR fluorophore IRDye 800CW via amine chemistry and intravenously dosed to evaluate in vivo targeting specificity. Increasing doses of imaging agent were given to estimate the saturating dose.

**Results:**

The NIR-labeled proteins successfully targeted colonic lesions in a murine model of colitis. Co-administration of a molar excess competing unlabeled dose reduced normalized uptake in diseased colon by > 70%. Near infrared ex vivo images of colon resected from diseased animals showed saturation at doses exceeding 1 nmol and was confirmed with additional quantitative ex vivo biodistribution. Cellular-level specificity and protein stability were assessed via microscopy.

**Conclusions:**

Our imaging data suggest the alternatively spliced D domain of tenascin C is a promising target for delivery-based applications in inflammatory bowel diseases.

**Supplementary Information:**

The online version contains supplementary material available at 10.1007/s11307-022-01758-6.

## Introduction

Inflammatory bowel diseases (IBDs) are progressive disorders of the gastrointestinal tract that hinder the patient’s quality of life if left untreated. Common intervention therapies include anti-inflammatory and immunosuppressive agents such as 5-amino salicylates for mild cases and corticosteroids in more severe cases [1, 2]. More recently, in light of the evidence for the crucial roles of cytokine production and signaling in IBD pathogenesis, biologic therapeutics have emerged with the potential to induce remission and heal the intestinal mucosa [2–4]. Unlike small molecule approaches, which can be formulated for tailored release via oral administration [5], larger molecular weight biologics often rely on parenteral dosing. These systemic treatments have yielded mixed results in mouse models and the clinic, where a lack of selective targeting and delivery has yielded low drug concentrations in the colon while accompanying adverse systemic responses [6–8].

Extracellular matrix (ECM) proteins are upregulated in a variety of inflammatory diseases due to increased tissue remodeling and have served as attractive imaging and drug delivery targets [9–12]. Increased ECM expression is well-documented in IBD, where remodeling is a consistent feature implicated in disease pathogenesis [13], and elevated serum levels of ECM proteins such as tenascin C are observed in IBD patients [14]. Tenascin C is a glycoprotein with multiple functional isoforms within the ECM. While some isoforms are expressed in healthy tissue, others such as isoforms containing domain D and A1 have been used in the clinic for delivery of radiolabeled antibodies due to their increased expression in tumor tissue [15–17]. Additionally, single-chain Fv (scFv) targeting tenascin C has been shown to specifically accumulate in tumors in several oncology models [18]. A related ECM target for delivery-based strategies, fibronectin extra-domain A, has seen promising transition from oncology to IBD and is well-reported [19]. However, tenascin C isoforms remain largely unexplored in IBD models, despite its reported expression. For example, in a commonly used murine colitis model, the addition of dextran sulfate sodium (DSS) to drinking water resulted in tenascin C upregulation in damaged mucosal areas [20]. Additionally, Kawamura et al. demonstrated a marked increase of both colonic protein and gene expression of tenascin C in a murine model of colitis-associated cancer [21]. Perhaps most notable, similar to the identification of fibronectin extra-domain A, in vivo protein biotinylation followed by liquid chromatography-tandem mass spectrometry also identified the alternatively spliced D domain of tenascin C (herein referred to as TNC D) in diseased mouse colon extracts [22–24]. Here, targeted molecular imaging can answer key questions involving disease expression, target engagement, and feasibility of locally delivered therapeutics [25–27].

Near infrared (NIR) imaging is increasingly used to elucidate the tissue-level distribution of various clinically relevant antibodies [28–31]. Not only is reagent generation time- and cost-effective, the lack of ionizing radiation and increased resolution make it an attractive modality for preclinical target identification. Despite these benefits, the overlap between NIR imaging and therapeutics development to evaluate novel antigens for targeted drug delivery is less common in IBD research. Instead, common colon therapeutic delivery methods include orally dosed nanoparticle formulations that rely on increased intestinal permeability or pharmacologically inactive prodrugs that require enzymatic catalysis [5, 32]. Due to incompatible labeling chemistries and risks of altering bioavailability—owing to the physicochemical properties of the detectable label—these delivery platforms often preclude molecular imaging for target engagement. Several positron emission tomography (PET) probes such as ^18^F-FDG for glucose metabolism and ^89^Zr-labeled cys-diabodies for CD4 + T cells have seen use in IBD research [33–36]. However, these probes focus on inflammation imaging, and the target expression is often not unique to colon. Thus, increasing the repertoire of antigens unique to diseased colon is fruitful, and molecular imaging can help identify and verify high expressing antigens. In this study, we generated novel antibody-based reagents specific to TNC D. Using immunohistochemistry (IHC) expression in clinical and preclinical colon samples as a guide, we evaluated TNC D as an in vivo target in preclinical murine IBD models using NIR imaging.

## Methods

### Antibody Affinity Maturation

Affinity maturation was performed via yeast display to generate high affinity anti-tenascin antibodies [37]. Libraries were designed by selective and limited mutagenesis based on predicted contact or hypermutation positions within CDRs of the antibody. Limited mutagenesis by polymerase chain reaction was performed using primers with low degeneracy at the targeted positions to create three antibody libraries in scFv format. The first library contained mutations at residues 30, 31, 33, 34, 50, 52, 52a, 54, 55, 56, and 62 in HCDR1 and HCDR2 (Kabat numbering) [38]; the second library at residues 95-100b in HCDR3; and the third library at residues 28, 30–32, 50, 53, 92–94, and 96 in LCDRs1-3. The three libraries were transformed into the EBY-100 strain of *Saccharomyces cerevisiae* as previously described [39], and diversity (~ 1 × 10^9^ per library) was estimated from serial dilution and plating on SD-UT agar plates. Each library was selected for improved affinity by binding against decreasing concentrations of biotinylated-TNC by magnetic-activated cell sorting (MACS) and fluorescence activated cell sorting (FACS). Yeast cells expressing scFv anti-TNC D antibody variants were allowed to bind for different times and temperatures before washing or addition of unlabeled competitor. All incubations and washes were in PBS containing 0.5% BSA. Bound biotinylated antigen was detected by flow cytometry using commercially available fluorescence-labeled streptavidin. Selection for improved on-rate, off-rate, or overall affinity was performed. Antibody sequences from selection rounds were recovered by Sanger sequencing and converted to recombinant IgG format for further characterization.

### Expression and Purification

Anti-TNC D IgG production is similar to previously described [10, 18]. The monoclonal antibody targeting TNC D was purified via ProteinA through mediated capture of the Fc domains of the IgG. The clarified supernatant was purified on a AKTAPure FPLC (Cytiva, Marlborough, MA) via ProteinA affinity capture using a HiTrap MabSelect SuRe column (Cytiva, Marlborough, MA) and eluted at low pH. A second purification was performed using a Superdex 200 pg (Cytiva, Marlborough, MA) size exclusion column using tris-buffered saline to yield > 95% protein purity as determined by analytical liquid chromatography (Agilent, Santa Clara, CA). Endotoxin level was quantified to be < 1 EU/mg (Charles River Laboratories, Wilmington, MA).

### Binding Assays

Binding kinetics for 800CW labeled and unlabeled anti-TNC D IgG against mouse-soluble TNC were measured via surface plasmon resonance on a BIAcore T200 instrument (GE Healthcare, Uppsala, Sweden) at 25 °C. Measurements for TNC D IgG were performed using Fc capture. Ten thousand RU of goat anti-human Fc polyclonal antibody (Thermo Fisher Scientific Inc., #31,170) was diluted to 20 µg/mL, respectively, in 10 mM sodium acetate (pH 4.5) and was immobilized on all four flow cells across CM5 chip via amine coupling followed by 1 M ethanolamine quench. Flow cell 1 was used as a reference. Chip preparation and binding kinetic measurements were made in the assay buffer HBS-EP + (10 mM HEPES, pH 7.4, 150 mM NaCl, 3 mM EDTA, 0.05% Tween 20). Assay cycles consisted of capture of the anti-TNC D IgG molecule at 1 µg/mL and a flow rate of 10 µL/min for 60 s followed by analyte injection of either murine tenascin or buffer control over both reference and active surface for 300 s at 50 µL/min, after which the dissociation was monitored for 15 min at 50 µL/min. Tenascin injections were flowed at increasing concentrations from 0.4 to 900 nM. Data were generated in triplicate and fitted globally to a 1:1 binding model using BIAcore T200 BioEvaluation software to determine the binding constants.

### Immunohistochemistry

Colons from C57BL/6 mice on 3% DSS in the drinking water for 7 days were either embedded in OCT or fixed in 4% paraformaldehyde (PFA) and sucrose-protected. OCT-embedded sections were acetone-fixed same day as sectioning and air-dried overnight. After sectioning, both acetone-fixed and PFA/sucrose-protected sections were brought to room temperature prior to IHC. Tissue sections were blocked with both dual endogenous block and protein block prior to a 30-min incubation with either anti-TNC D or isotype IgG at 0.03 µg/mL. The primary human antibodies were detected sequentially with rabbit anti-human IgG (Southern Biotech, Birmingham, AL), HRP polymer, and visualized with DAB with a hematoxylin counterstain (Leica Bond Polymer Refine Detection kit). Following immunolabeling and detection, the glass slides were imaged using a Pannoramic 250 whole slide scanner at 20 × magnification (3D Histech, Budapest, Hungary). To visualize the NIR signal at a microscopic scale, 5-µm colon sections were counterstained with Hoechst 33,342 (Invitrogen, Waltham, MA) and an AlexaFluor 647 labeled anti-human IgG (Southern Biotech, Birmingham, AL) and imaged using a Leica DMi8 fluorescent microscope equipped with 405-nm, 635-nm, and 740-nm LEDs.

Clinical IBD colon was evaluated using a biotinylated anti-TNC D antibody. Frozen human tissue was obtained from CHTN (National Cancer Institute, Bethesda, MD), Folio Biosciences (Columbus, OH), and UMASS Memorial Gastroenterology (Worcester, MA). Frozen human sections were post-fixed with acetone on the day of sectioning and air-dried overnight. Fixed sections were brought to room temperature prior to IHC. Tissue sections were blocked with dual endogenous block, a streptavidin/biotin blocking kit (Vector Labs, Burlingame, CA), and a protein block, followed by a 15-min incubation with human anti-TNC D IgG at 0.7 µg/mL. The biotin-labeled, anti-TNC D IgG was detected using Vectastain Elite ABC-HRP reagent (Vector Labs, Burlingame, CA) and visualized with DAB with a hematoxylin counterstain (Leica Bond Polymer Refine Detection kit). Slides were scanned on a Pannoramic 250 slide scanner.

### IRDye 800CW Conjugation

Solutions of anti-TNC D IgG or isotype IgG (human IgG1 recognizing human cytomegalovirus) were buffered exchanged to PBS using 7-kDa MWCO Zeba desalting columns. To these protein solutions (50 nmol in 1 mL), 100 µL of 7.5% sodium bicarbonate was added. The mixture was mixed by inverting before addition of IRDye 800CW NHS ester (40 nmol in DMSO). The solution was mixed again by gently inverting and reacted at room temperature for 2 h. Unreacted and hydrolyzed fluorophore was removed using two Zeba desalting columns (7-kDa MWCO). Fluorophore degree of label was determined using a NanoDrop 2000c spectrophotometer (ThermoFisher Scientific, Waltham, MA). Monomer purity was determined via SEC-HPLC with 280-nm and 770-nm detection.

### Animals

Rodent studies were conducted at AbbVie to the standards of the Association for the Assessment and Accreditation of Laboratory Animal Care (AAALAC) standards. All studies with mice were performed according to approved protocols by AbbVie’s Institutional Animal Care and Use Committee (IACUC). Briefly, 7-day acute murine colitis was induced in wild-type, 12-week-old, female C57BL/6 mice (Taconic Biosciences, Rensselaer, NY) with 3% DSS (MP Biomedicals, Santa Ana, CA) in the drinking water. Mice body weights were monitored, and animals were euthanized if > 20% of the initial weight was lost. To evaluate in vivo targeting specificity, animals were intravenously dosed with 1 nmol of anti-TNC D 800CW with or without 20 nmol of competing unlabeled anti-TNC D on day 7 of DSS treatment. To estimate the in vivo saturating dose, a separate cohort of animals was intravenously dosed with increasing doses of anti-TNC D IgG-800CW ranging from 0.1 to 10 nmol. To determine the blood clearance rates for the fluorescent proteins, mice were intravenously dosed via the tail vein on day 7 of DSS. At predetermined timepoints, 20 µL of whole blood was collected via a tail nick, mixed with 80 µL of 10 mM EDTA in PBS, and frozen at − 80 °C. The samples were later thawed, and 800CW fluorescence was measured using an Odyssey CLx (Li-Cor, Lincoln, NE). Fluorescence was converted to whole blood concentration using a standard curve to quantify the absolute concentration of fluorescent protein in the blood similar to previously published methods [40]. The clearance was fit to a biexponential decay. Fitted initial concentration was compared with theoretical values based on dose and total blood volume. After the last blood time point, animals were euthanized and organs resected and visualized using a Pearl Imager (Li-Cor, Lincoln, NE). Higher resolution colon images were collected using the Odyssey CLx.

### Ex Vivo Biodistribution

The following protocol was modified from previously published protocols for quantifying uptake of NIR-labeled fluorescent proteins ex vivo [41, 42]. Following the last blood draw, mice were euthanized, and organs were resected and imaged on a Pearl Imager. Organs were weighed, and incubated at 37 °C in a mixture of RIPA buffer in PBS supplemented with 6 mg/mL collagenase type IV for 30 min. An equal volume of 0.25% trypsin/EDTA was added, and the solution was incubated for another 30 min before being homogenized using a FB-120 Sonic Dismembrator. Known amounts of digested NIR conjugates were used to generate a calibration curve, and bulk signal from organ digests was converted to absolute concentrations to quantify the %ID/g.

### Histology

Colons were excised, and the distal 5 cm was cut into segments. Samples were fixed in 10% neutral buffered formalin for 24 h, processed, and embedded in paraffin wax, before being sectioned at 5 μm and stained with hematoxylin and eosin (H&E). Slides were scanned using a Pannoramic 250 whole slide scanner at 20 × , and erosion length measurements were measured using Visiopharm software (Visiopharm, Denmark).

## Results

**Fig. 1:**
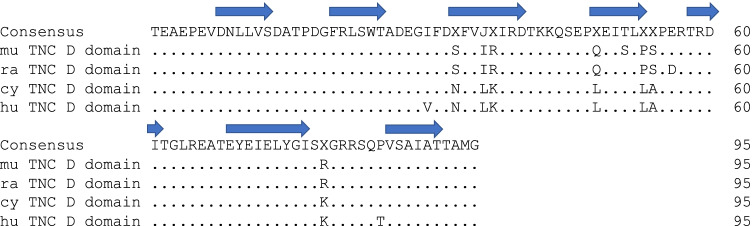
Sequence alignment for TNC D domains from mouse, rat, cynomolgus monkey, and human. Domain annotations derived from available tenascin C FNIII structure (PDB ID: 1TEN).

**Fig. 2:**
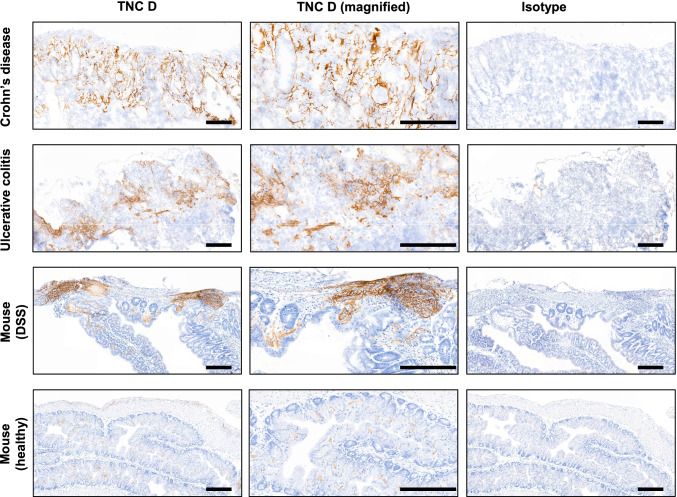
Immunohistochemistry panel for TNC D in colon. Target validation in sections of human and animal colon tissue with antibodies specific to the alternatively spliced D domain of tenascin C. Scale bars, 200 µm.

Sequence alignment comparison for TNC D between human, rat, cynomolgus monkey, and mice shows conservation across human and model organisms (Fig. 1). Affinity maturation via yeast display yielded an antibody with high affinity to TNC D. Surface plasmon resonance determined the affinity to be single-digit nanomolar (*K*_*D*_ = 1.4 nM) to murine TNC D (Table 1). Comparable decrease in affinity was observed after fluorophore conjugation (*K*_*D*_ = 1.4 nM vs *K*_*D*_ = 2.1 nM for unlabeled and labeled, respectively). TNC D is expressed in the lamina propria of patients with Crohn’s disease and ulcerative colitis (Fig. 2). In the preclinical mouse colitis model, staining is observed at the site of lesions throughout the lamina propria to the muscularis mucosae. This staining pattern is not observed in healthy samples, where reduced expression can be found in the superficial lamina propria, in proximity to mucosal epithelial cells.

**Table 1:** Surface plasmon resonance determining the affinity to be single-digit nanomolar (*K*_*D*_ = 1.4 nM) to murine TNC D

Reagents	*k*_on_ (M^−1^ s^−1^)	*k*_off_ (s^−1^)	*K*_D_ (M)
Anti-TNC D IgG	2.7 × 10^5^	3.7 × 10^−4^	1.4 × 10^−9^
Anti-TNC D IgG-800CW	2.0 × 10^5^	4.3 × 10^−4^	2.1 × 10^−9^

**Fig. 3:**
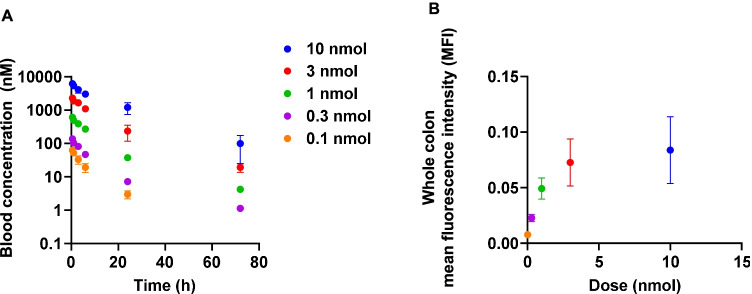
**A** Blood clearance for intravenously dosed anti-TNC D IgG-800CW suggests linear pharmacokinetics for imaging doses. **B** Whole-organ mean NIR fluorescence intensity increases with dose. Mean fluorescence intensities approach saturation beyond a 1 nmol dose.

**Fig. 4:**
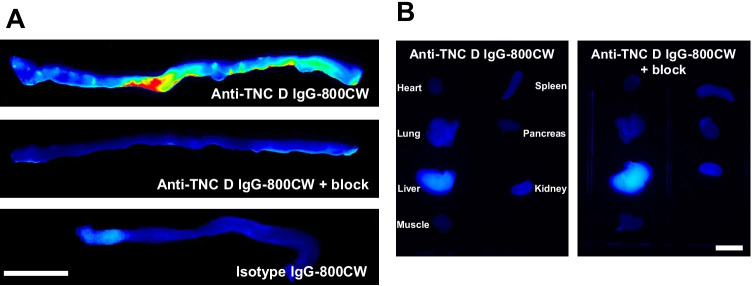
**A** From top to bottom: anti-TNC D IgG-800CW (1 nmol) in DSS-treated colon; anti-TNC D IgG-800CW (1 nmol) + anti-TNC D IgG (20 nmol) in DSS-treated colon; isotype IgG-800CW (1 nmol) in DSS-treated colon. Macroscopic organ images show increased NIR signal for anti-TNC D IgG-800CW. Reduction in signal observed in the blocking and isotype IgG groups. **B** Anti-TNC D IgG-800CW (left) and anti-TNC D-800CW + blocking (right). No differences in uptake are observed in non-target organs. Scale bars, 10 mm.

Anti-TNC D IgG was labeled with the NIR fluorophore IRDye 800CW to evaluate in vivo tenascin targeting in a murine DSS colitis model. As the absolute TNC D expression is unknown in this model and a goal was to identify the saturating dose through a wide range of doses, the pharmacokinetics of the NIR-labeled antibody was thoroughly characterized. Systemic clearance of the NIR-labeled proteins exhibited strong linear pharmacokinetics for doses ranging from 0.1 to 10 nmol in diseased animals (Fig. 3A). An average fluorophore degree of label below 1 was used to minimize the impact of the dye on the pharmacokinetics of the antibody and intramolecular quenching. Fitted initial blood concentrations were within reasonable values for all doses. The clearance data were fit to a biexponential decay with distribution and clearance phase half-lives calculated (Table 2). At each dose, the animals were euthanized after 72 h and the colon resected for macroscopic imaging. Whole organ mean fluorescence intensities (MFI) captured using a Pearl Imager were measured and plotted against dose to identify target saturation (Fig. 3B). Average colon MFI suggests the saturating dose to be between 1 and 3 nmol (0.05 ± 0.1 vs 0.07 ± 0.02 for 1 and 3 nmol, respectively, *P* = 0.15). With this dose in mind, a representative image for diseased colon from mice dosed with 1 nmol anti-TNC D IgG-800CW is shown (Fig. 4A, top). Here, increased NIR signal is seen at the site of lesions. To further evaluate in vivo specificity, 20 nmol of unlabeled anti-TNC D IgG was co-dosed with 1 nmol of anti-TNC D IgG-800CW (Fig. 4A, middle). A significant reduction in signal was observed with molar excess unlabeled IgG block. Dosing a NIR-labeled IgG1 isotype yielded similar results as the blocked group (Fig. 4A, bottom). Visually, the blocking dose had minimal impact in the remaining organs (Fig. 4B). Analogous colon imaging data were collected in untreated, healthy controls (Fig. S5). In these healthy groups, the lowest macroscopic colon signal was observed, suggesting no detectable TNC D targeting, and this agreed well with IHC (Fig. 2). In these healthy animals, addition of an unlabeled blocking dose did not reduce average colon signal any further.

**Table 2:** Distribution and clearance phase half-lives

Group	Fraction fast	*t*_1/2,α_ (h)	*t*_1/2,β_ (h)
Anti-TNC D IgG-800CW	0.7 ± 0.1	4 ± 1	14 ± 3

**Fig. 5:**
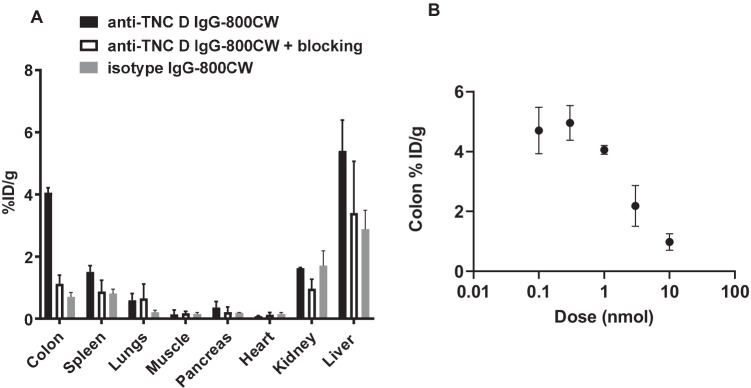
**A** Ex vivo biodistribution for DSS-treated animals dosed with either 1 nmol anti-TNC D IgG-800CW or 1 nmol anti-TNC D IgG-800CW + 20 nmol unlabeled anti-TNC D IgG 72 h post-IV. Whole-organ homogenate shows higher uptake of the anti-TNC D IgG in colon. **B** Whole colon ex vivo biodistribution for DSS-treated animals dosed with increasing doses of anti-TNC D IgG-800CW 72 h post-IV. Increasing the dose beyond 1 nmol decreases normalized uptake, suggesting saturation.

As the imaging data suggested 1 nmol was sub-saturating (though nearing saturation), the organs from this group were then weighed, homogenized, and scanned with digest standards to quantify normalized uptake (Fig. 5A). In agreement with the imaging results, blocking with 20 nmol of unlabeled anti-TNC D IgG reduced total colon uptake (4.1 ± 0.2 vs 1.1 ± 0.3%ID/g for w/o and w/ blocking dose, respectively, *P* < 0.0001). No significant differences in uptake were observed in spleen, lung, pancreas, heart, and muscle between dosed groups. Increased normalized uptake was observed in the liver for all dosed groups, although there was no significant difference between the groups (5 ± 1 vs 3 ± 2 for no block and block, respectively, *P* = 0.24). This is likely due to a combination of increased negative charge density due to fluorophore conjugation and the residualizing nature of the fluorophore and not due to specific targeting [40, 43, 44]. To corroborate the colon saturation data obtained via imaging, normalized uptake values were also calculated using the homogenized colons across all dosed groups. A maximum and near constant %ID/g was observed for 0.1, 0.3, and 1 nmol doses (4.7 ± 0.8 vs 5.0 ± 0.6 vs 4.1 ± 0.2%ID/g for 0.1, 0.3, and 1 nmol doses, respectively, *P* = 0.2). However, as dose increased to 3 and 10 nmol, %ID/g decreased from 2.2 ± 0.7 to 1.0 ± 0.3, suggesting in vivo target saturation. With both ex vivo macroscopic imaging and quantitative ex vivo biodistribution data converging on a similar saturating dose, we attempted to estimate local target density using the 1 nmol dose data (SI). Subtracting the blocked dose from experiments without blocking, specific uptake was estimated at 3%ID/g for the near saturating 1 nmol dose at 72 h, or > 20 nM for the average colon concentration. Using estimated number of observed lesions in the diseased colon along with estimates of lesion size (Fig. S3), corrections for lesion volume fraction likely results in a higher local antigen density (SI).

**Fig. 6:**
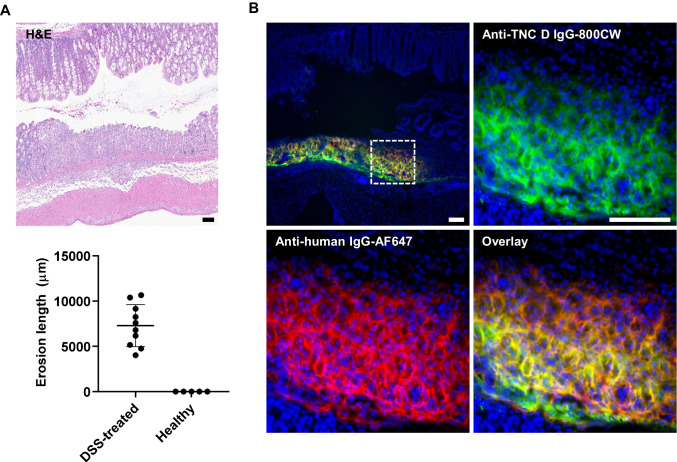
**A** From top to bottom: diseased murine colon H&E showing immune cell infiltrate; erosions that are present in diseased animals and absent in healthy mice are defined as skip lesions on both sides of the colon sections and are characterized by areas with no epithelium on top of the mucosa. Each data point represents the average lesion length for one animal. **B** Fluorescent microscopy for cellular-level distribution of NIR anti-TNC D IgG in diseased mouse colon. Cell nuclei are stained with Hoechst 33342 (blue); anti-TNC D IgG-800CW (green); anti-human-AF647 IHC (red). Scale bars, 100 µm.

Lastly, histology confirmed cellular-level disease activity, where heavy immune cell infiltrate was observed at the site of colonic lesions in mice provided DSS in drinking water (Fig. 6A). Thus, one concern for molecular imaging of extracellular expressed tenascin C was degradation of the bound antibody via protease degradation or phagocytosis. Unlike internalizing cell surface receptor targets, which can provide specific, residualizing fluorescence signal, ECM imaging relies on slow dissociation kinetics/high antigen expression and intact targeting antibody to mitigate non-specific signal. Co-localization between the human anti-TNC D NIR signal from IV dosed protein and IHC using an anti-human IgG-AF647 suggests minimal metabolism and trafficking of the NIR label in diseased colon (Fig. 6B).

## Discussion

Increased expression of ECM components such as fibronectin and tenascin C in cancer and autoimmune diseases is well-documented [24, 45, 46]. While in vivo targeting of fibronectin extra-domain A is well-characterized for molecular imaging and drug delivery in various autoimmune diseases including IBD [19], the previously identified D domain of tenascin C is largely unexplored for gastrointestinal diseases. In addition to sequence alignment showing strong species conservation for TNC D across models, our IHC pointed to unique expression in the lamina propria in clinical IBD colon biopsies and in colonic lesions of the commonly used DSS mouse colitis model. Thus, a monoclonal antibody with nanomolar binding affinity to TNC D was produced and evaluated in a murine colitis model via molecular imaging.

Near infrared targeted molecular imaging has played an increasingly important role in preclinical research, ranging from pharmacokinetics to elucidating tissue and cellular-level distribution of proteins and low molecular weight molecules [47, 48]. In this work, proteins were labeled via amine chemistry with IRDye 800CW to characterize systemic clearance and verify colon specificity in a murine colitis model. Macroscopic organ NIR images revealed punctate lesions ranging from the distal to proximal end of the colon. As targeted molecular imaging for TNC D is not reported in the literature and given the prevalent use of the mouse DSS colitis model in preclinical research, we sought to provide multiple experimental approaches to characterize target saturation. First, macroscopic image analysis for organ-level MFI was generated. Here, increasing doses of NIR-labeled imaging agent were dosed until images appeared saturated. Due to difficulties such uncertain detector linearity for the NIR camera and difficulty in generating imaging standards for concentration data conversion, a more laborious ex vivo biodistribution dataset was generated based on published full organ digest protocols [41, 42]. With a saturating dose identified between 1 and 3 nmol, in vivo saturable binding was further confirmed using a high molar blocking dose and this specificity for TNC D was further corroborated by IHC and NIR microscopy.

Empirically, extracellular receptors have some of the highest contrast values for imaging agents, and delivery pathways of therapeutic proteins—such as ADCs—share similarities with imaging agent delivery. However, a distinction for IBD in immunological targets is often soluble or surface expressed in subsets of motile cells [49, 50]. Therefore, targeted IBD therapeutics of local cytokine delivery should avoid internalization pathways for the targeting ligand. This makes ECM targets attractive, where a combination of high local ECM expression and protein affinity can drive preferential accumulation in disease following pharmacokinetic clearance. Here, slow dissociation due to *k*_off_ kinetics post-washout would ideally drive target-mediated efficacy. Unlike cell surface-expressed receptors, where probe design steps can be taken to improve internalization and residualization of the targeting ligand to mitigate poor in vivo expression, delivery of imaging agents to ECM targets largely relies on engineering stronger affinity to the target and/or faster blood clearance for efficient delivery and rapid washout. However, screening for ultra-high affinity binders (sub-nanomolar) risks non-specific binding and altering blood clearance may prove challenging without significant changes in protein format. Thus, one practical alternative is to identify ECM targets with high absolute local expression unique to disease tissue. The murine blocking experiments in this work estimate absolute TNC D density at the site of lesions to be higher than 200 nM assuming a conservative estimate of lesion volume fraction in the diseased colon. While protocols are more established for quantifying in vivo cell surface receptor density such as organ digests followed by flow cytometry [51, 52], antigen density measurements for ECM are challenging. There are caveats to the estimates provided in this work, such as assumptions lesion size, number, and expression across animals and minimal delivery limitations such as tissue diffusion and vascular extravasation. With this in mind, the estimates presented here are similar to cell surface molecular imaging targets present in autoimmune diseases such as type 1 diabetes and various cancers with high reported imaging target to background ratio and clinical success [42, 53, 54].

## Supplementary Information

Below is the link to the electronic supplementary material.

Supplementary file1 (DOCX 20372 KB)

## Data Availability

The datasets generated during and/or analyzed during the current study are available from the corresponding author on reasonable request. **Ethical Approval.** All applicable institutional and/or national guidelines for the care and use of animals were followed.
