# Supplementary material for: Imaging the Alternatively Spliced D Domain of Tenascin C in a Preclinical Model of Inflammatory Bowel Disease
Source: Mol Imaging Biol. 2022 Jul 29;25(2):314–23. doi: 10.1007/s11307-022-01758-6 (PMC10006278; doi:10.1007/s11307-022-01758-6)
Supplement: Supplementary file 1 — Supplementary file1 (DOCX 20372 KB) [file 11307_2022_1758_MOESM1_ESM.docx]

**Supplementary information**

**Imaging the Alternatively Spliced D Domain of Tenascin C in a Preclinical Model of Inflammatory Bowel Disease**

**Table of Contents**

1. Reagent characterization

2. TNC D local density estimate

3. Imaging controls

**1. Reagent characterization**


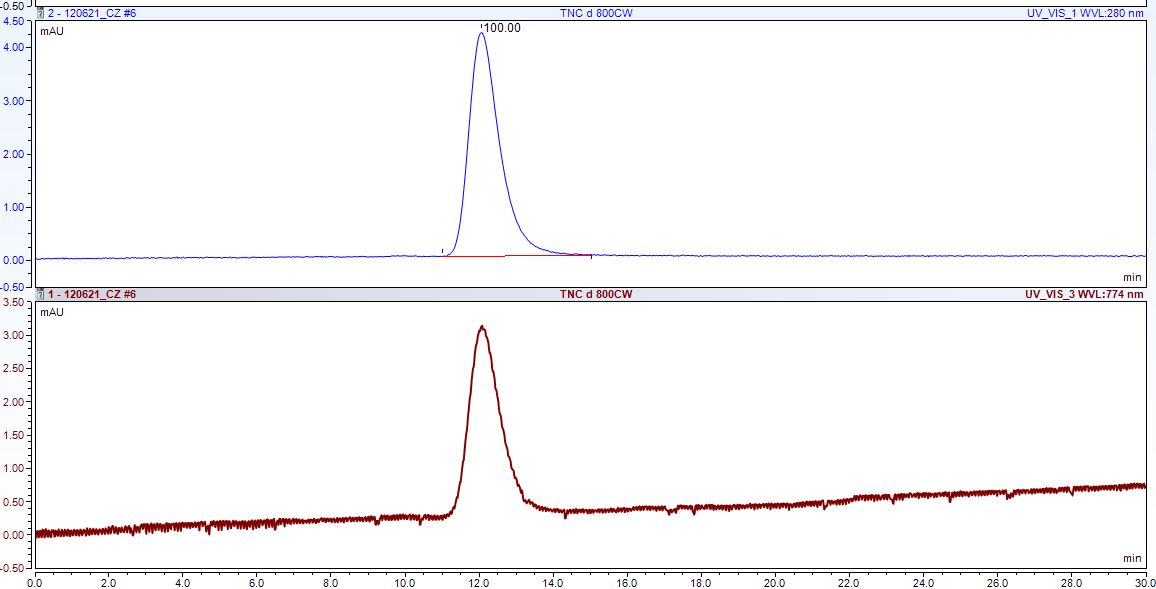


**Figure S1.** Size exclusion chromatograms for anti-TNC D IgG-800CW with 280 nm (top) and 774 nm (bottom) absorption. The data suggest high monomer purity and minimal unreacted fluorophore.

**
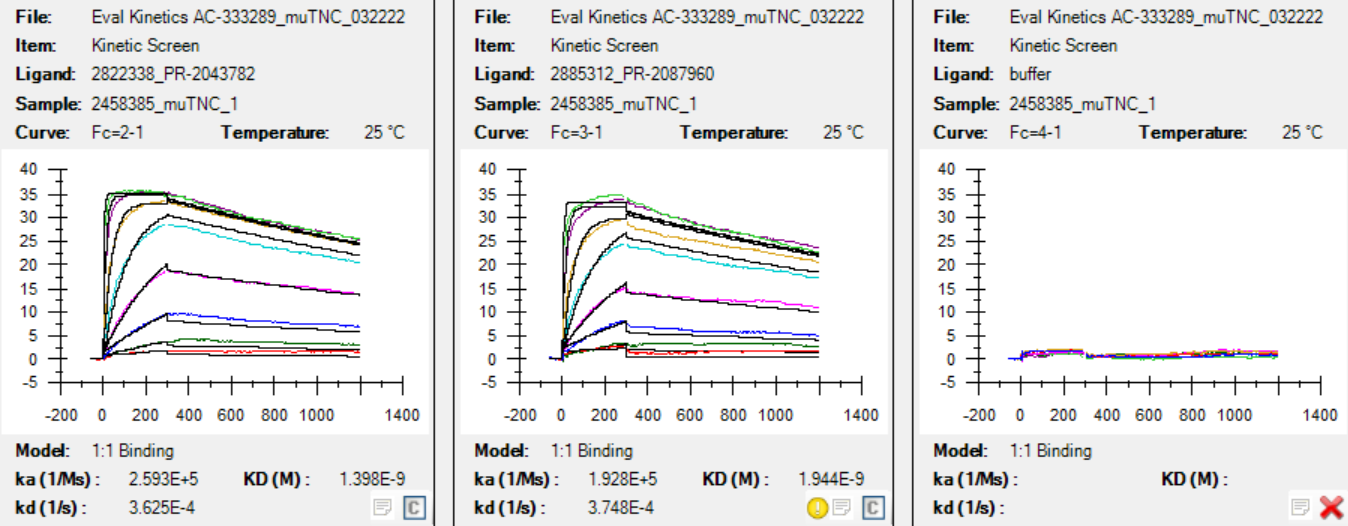
**

**Figure S2.** Representative surface plasmon resonance (SPR) sensorgrams for unlabeled (left) and 800CW labeled (middle) TNC D IgG and buffer control (right); data were collected and reported in triplicate. The data suggest minimal impact of binding to TNC D post-fluorophore conjugation.

**2. TNC D local density estimate**

**
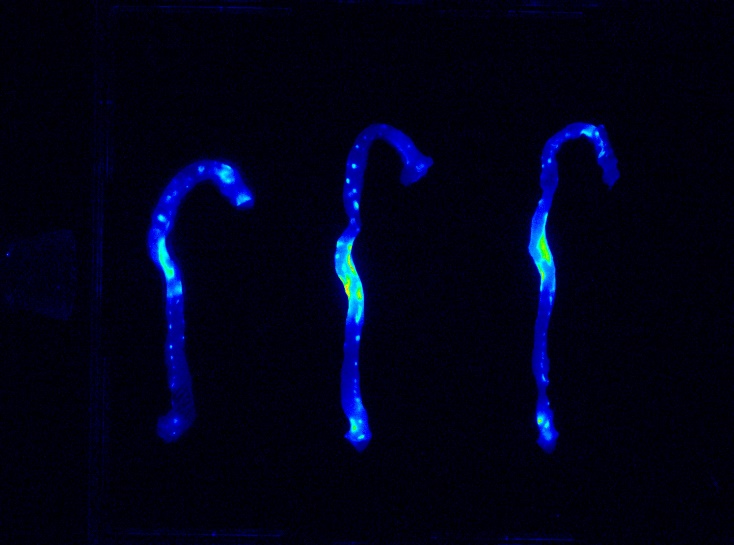
**


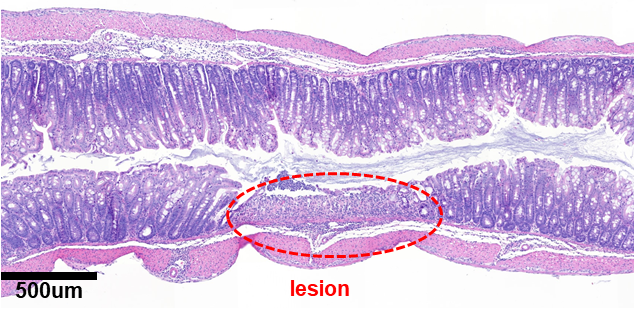


**Figure S3.** Mouse colon (H&E, left). A colonic lesion is circled (red). From the H&E and macroscopic NIR image (right), lesions appear to be ellipsoid in shape with minor and major axes averaging 100 µm and 300 µm, respectively. On average, we observe between 20-30 lesions per colon with an estimated average volume of 4 x 10^-5^ cm^3^.

Using a near saturating dose of high affinity (K_D_ ~ 2 nM) anti-TNC D IgG, colon digest data were obtained from 1 nmol dosed group. The specific %ID/g is calculated to be 3 %ID/g or an organ average > 20 nM. Depending on lesion volume fraction the local concentration may vary multiple fold. For example, 2 %V/V would result in 1000 nM local target density whereas a more conservative 10% would equate to 200 nM target density.


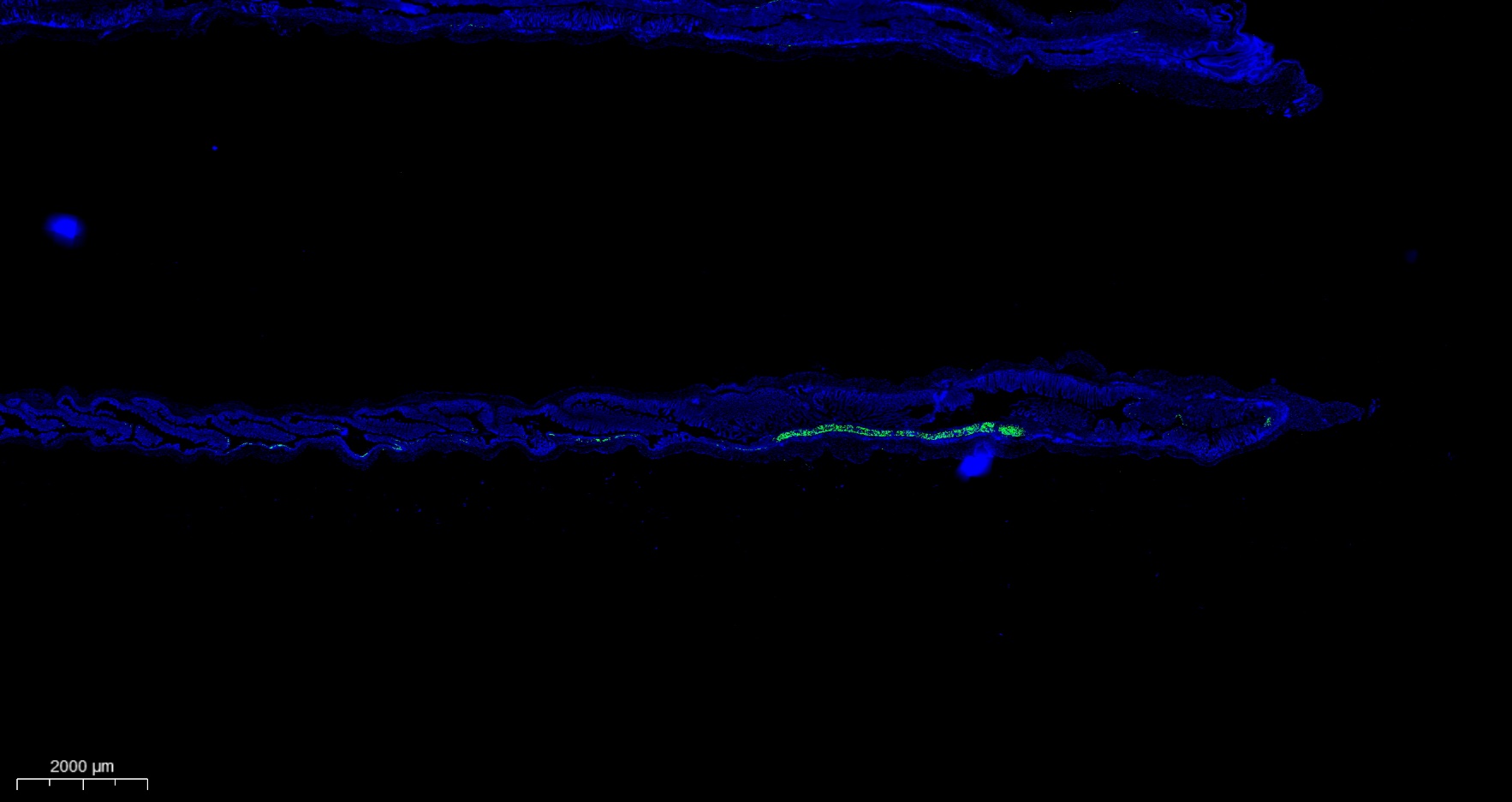

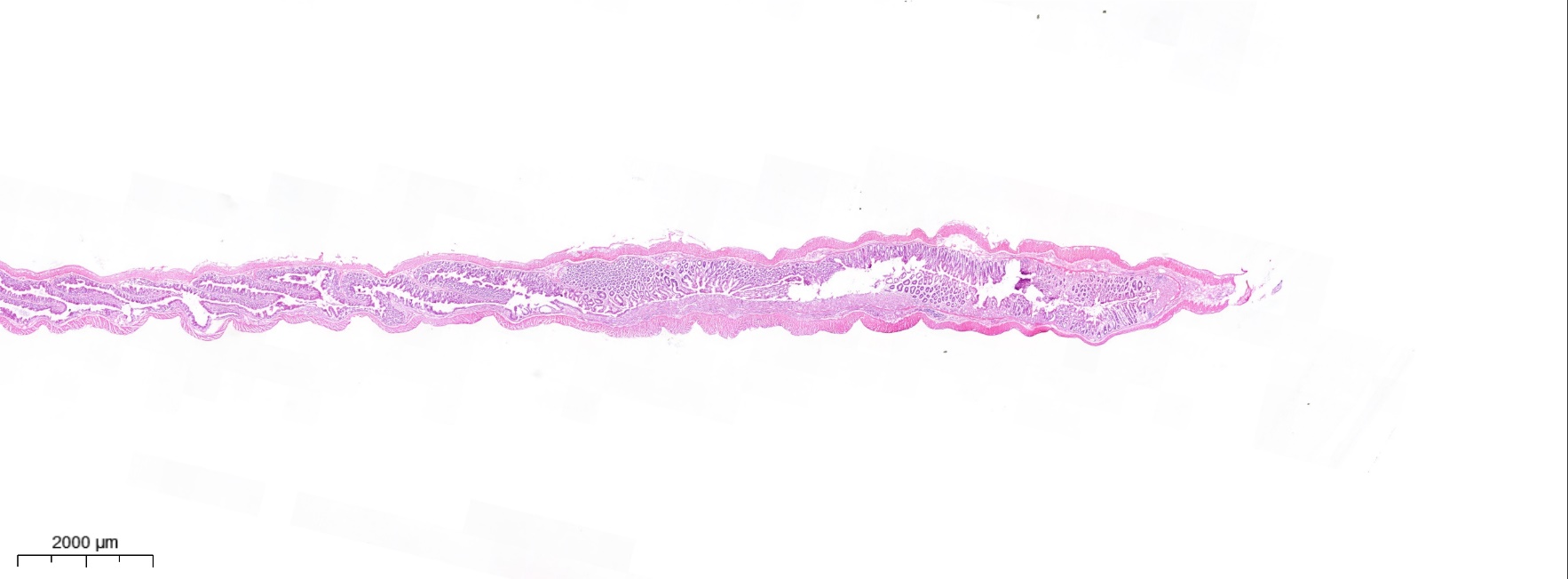


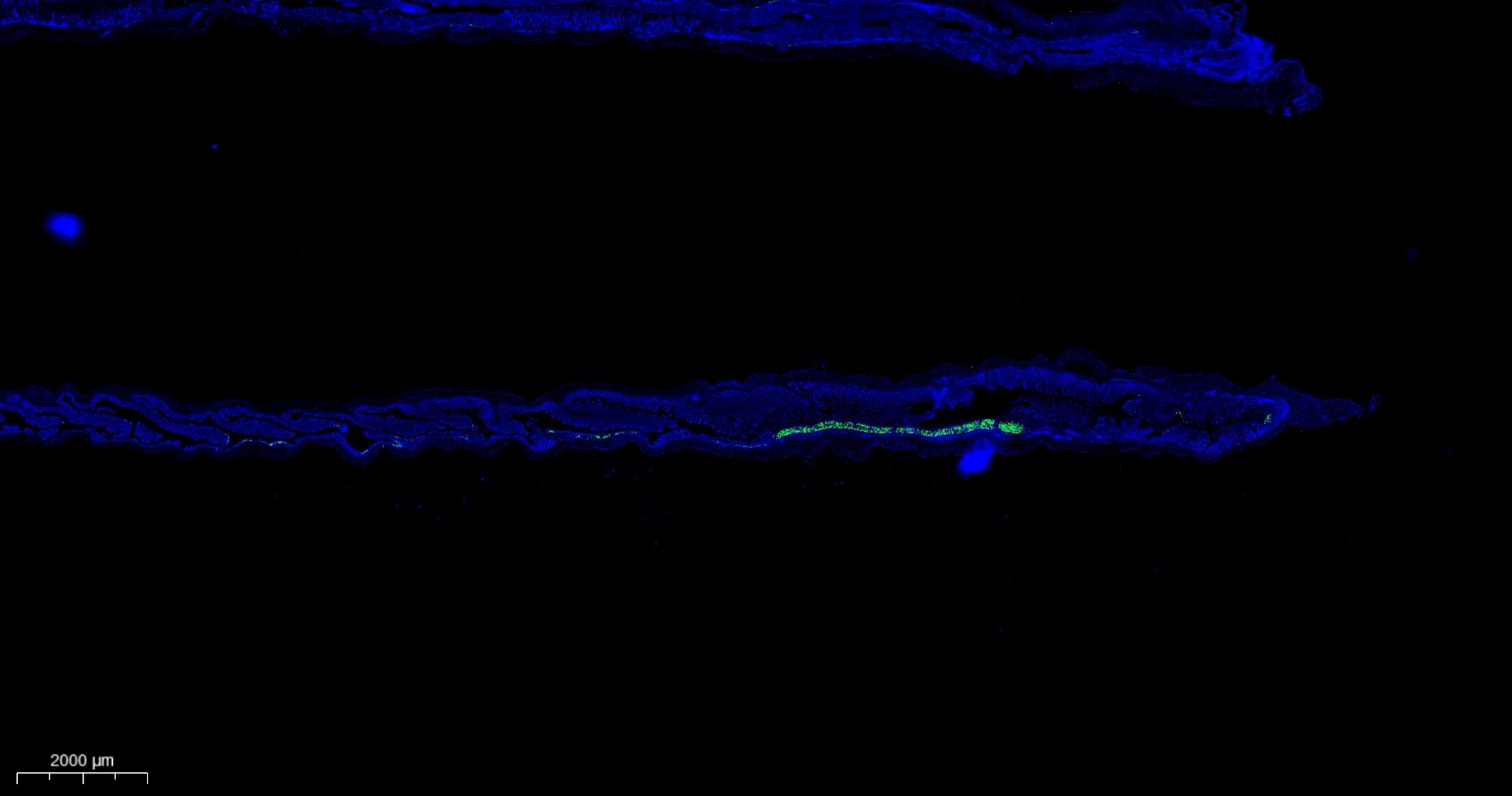


**
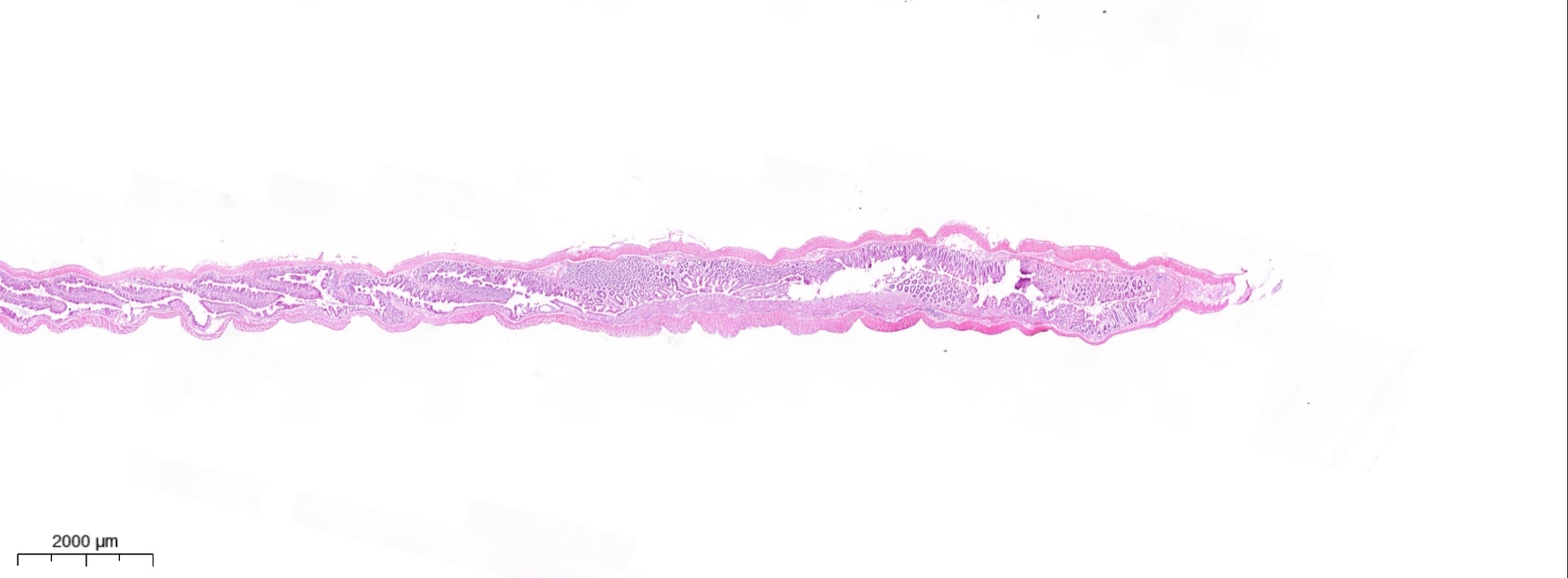
**

**Figure S4.** Mouse colon whole section (H&E + anti-TNC overlay top, anti-TNC middle, H&E bottom). While complete overlays are challenging for H&E and fluorescence, the serial section scans for fluorescence and H&E suggest the fluorescence signal is unique to colonic lesions.

**3. Imaging controls**

**
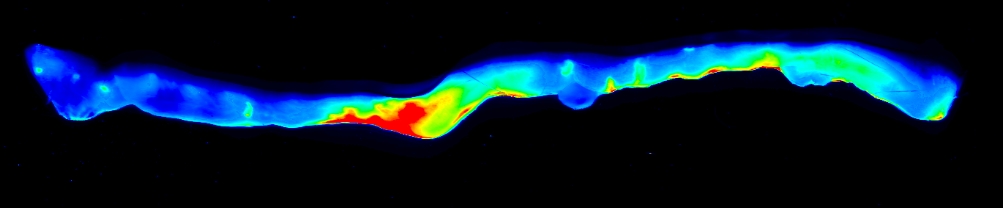

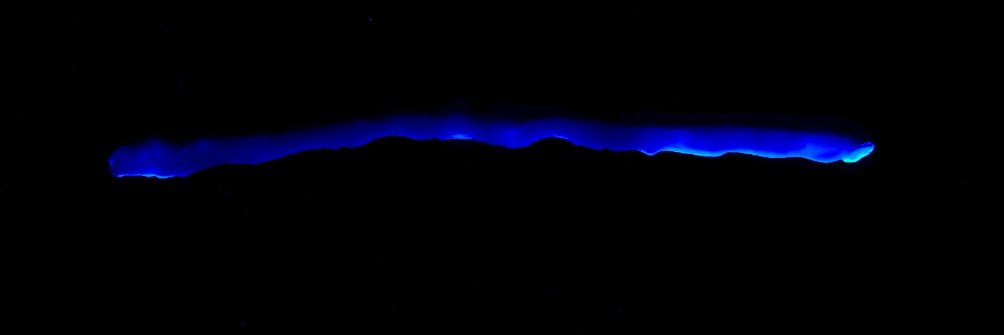

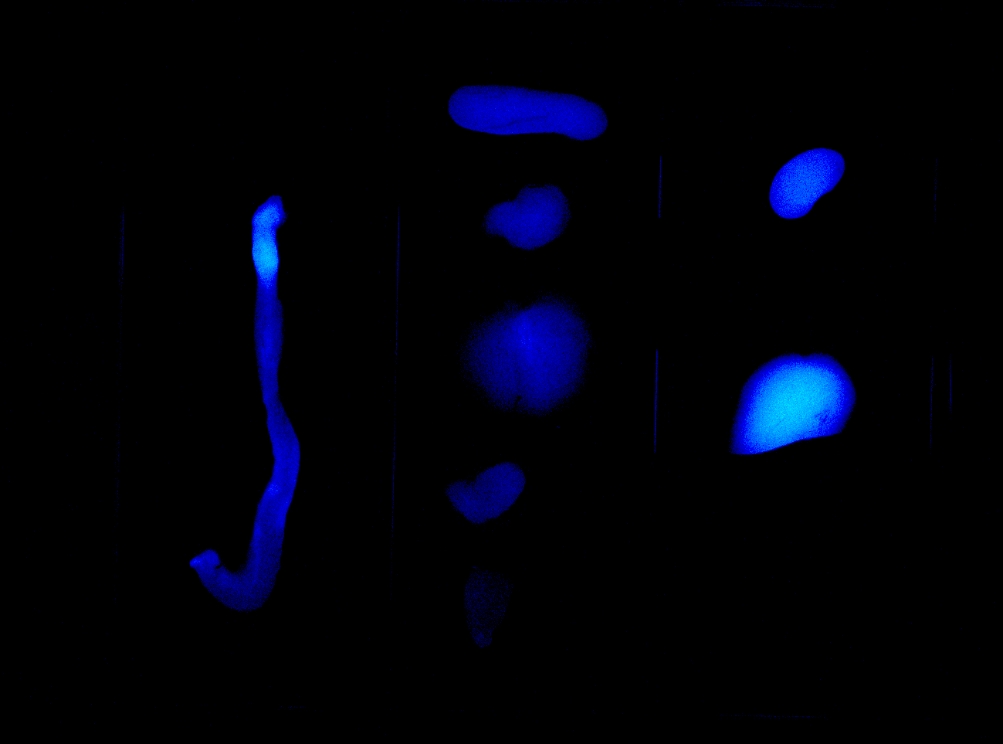

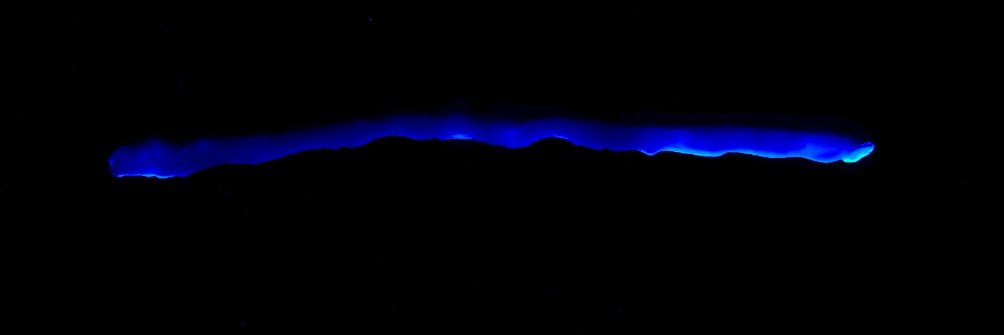

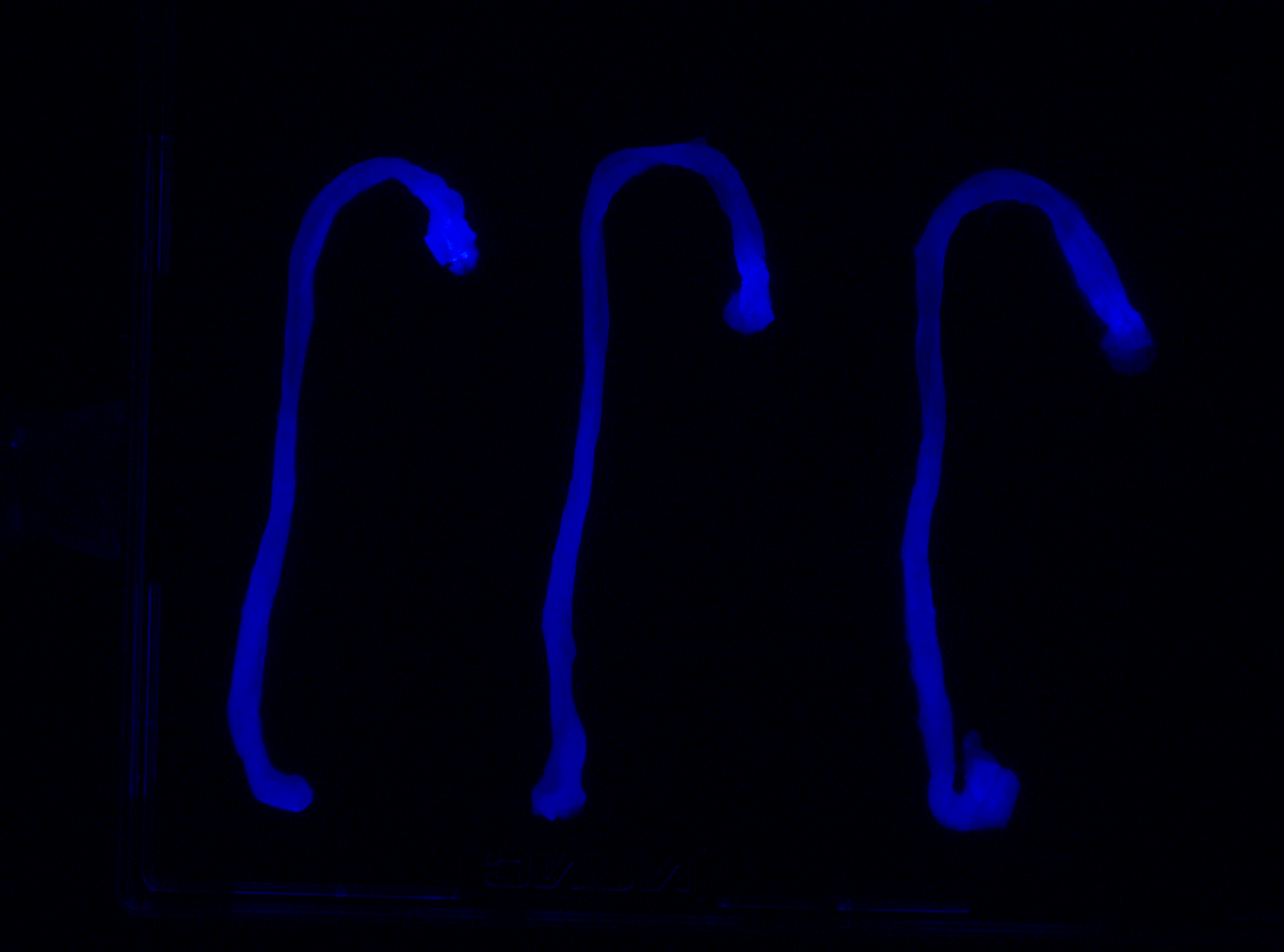

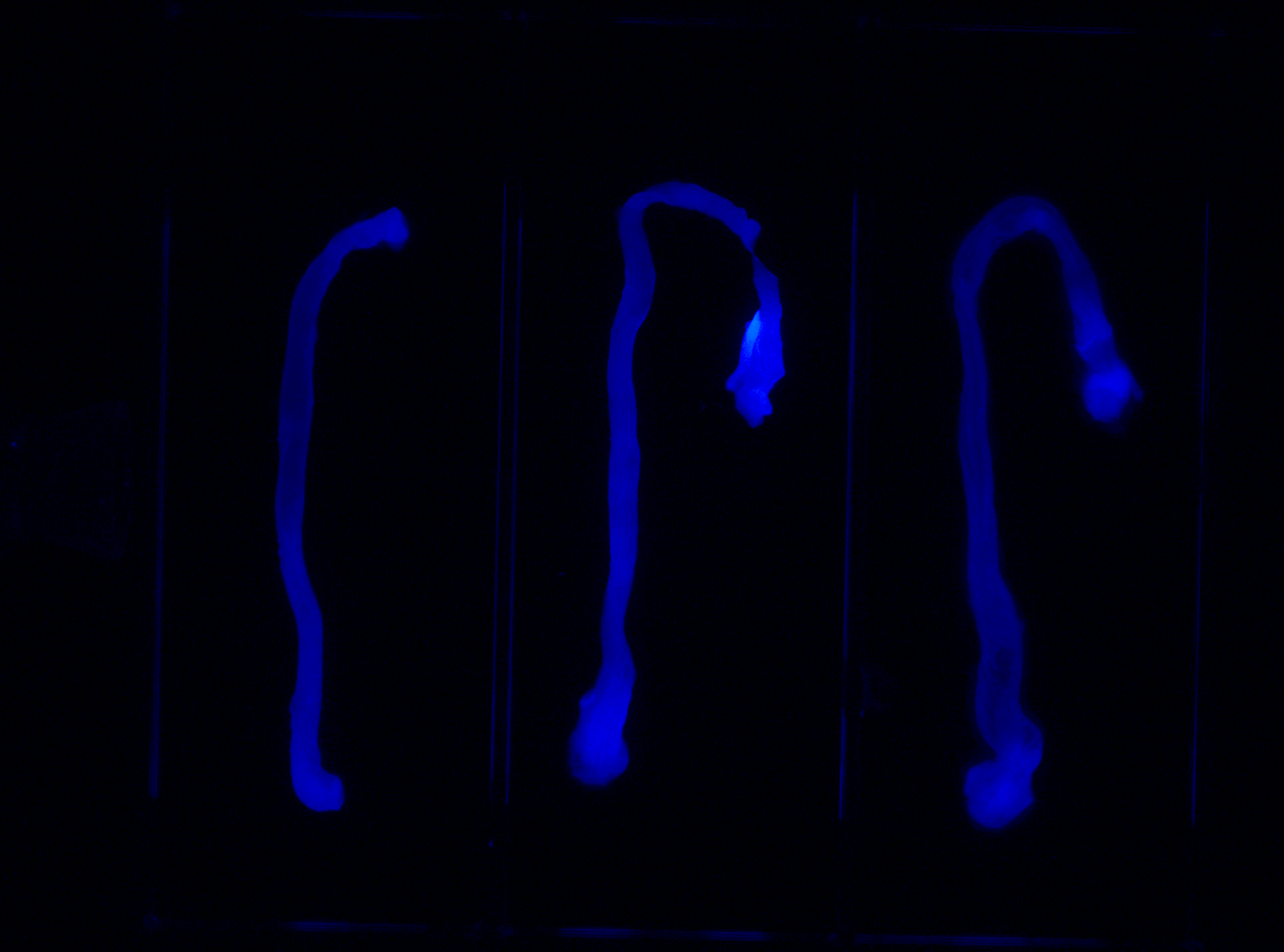
**

**Figure S5.** From top to bottom: anti-TNC D IgG-800CW (1 nmol) in DSS-treated colon; anti-TNC D IgG-800CW (1 nmol) + anti-TNC D IgG (20 nmol) in DSS-treated colon; isotype IgG-800CW (1 nmol) in DSS-treated colon; anti-TNC D IgG-800CW (1 nmol) in healthy colon; anti-TNC D IgG-800CW (1 nmol) + anti-TNC D IgG (20 nmol) in healthy colon. Macroscopic organ images show increased NIR signal for anti-TNC D IgG-800CW. Reduction in signal observed in the blocking and isotype IgG groups. Lowest signal observed in healthy groups with or without the blocking dose. Scale bars, 10 mm.
